# Oral problems and associated risk indicators in adults in the Russian Federation, India, and China

**DOI:** 10.1186/s12903-019-0811-8

**Published:** 2019-06-14

**Authors:** Rahul Bawankule, Abhishek Singh, Kaushalendra Kumar, Sarang Pedgaonkar

**Affiliations:** 1 International Institute for Population Sciences, Mumbai, 400088 India; 2 Department of Public Health and Mortality Studies, International Institute for Population Sciences, Mumbai, India; 3 Department of Population Policies and Programme, International Institute for Population Sciences, Mumbai, India

**Keywords:** Oral problems, The Russian Federation, India, China, BRICS, SAGE

## Abstract

**Background:**

Oral problems, known as a neglected epidemic, have become prevalent in Brazil, the Russian Federation, India, China, and South Africa (BRICS) countries in last decade. The objective of the study is to examine the prevalence and associated risk indicators of oral problems in adults in the Russian Federation, India, and China in BRICS countries.

**Methods:**

We used data from the first round of the Study of Global AGEing and Adult Health (SAGE), conducted by WHO in 2007–10 in selected BRICS countries. Oral problems are defined as if an adult had any mouth and/or teeth related problems including swallowing problems in last 1 year of the survey. We estimated the mean age of adults who had oral problems and used a t-test for comparing it by sex of adults. We determined the prevalence of oral problems in adults. We designed a hierarchical conceptual model to identify associated risk indicators with oral problems. Finally, we applied a multivariable binary logistic regression model based on a conceptual model to examine associated socioeconomic and demographic, behavioral and nutritional risk indicators and systemic diseases – diabetes, hypertension, and angina pectoris/angina with oral problems in adults.

**Results:**

The mean age of adults who had oral problems is lowest in India (57 years; SD: 15) and highest in China (65 years; SD: 11). However, it does not vary by sex of adults except India. The prevalence of oral problems is highest in the Russian Federation (35%) and lowest in China (9%). Adults with body mass index (BMI) less than 25 kg/m^2^, age 45 years or more, diabetes, hypertension, and angina pectoris/angina have a higher risk of oral problems. Females and adults using alcohol are also more likely to have oral problems in selected countries.

**Conclusions:**

The study concludes that females, adults using alcohol and those having any systemic disease are at higher risk of oral problems in the Russian Federation, India, and China. A one-third of adults had oral problems in particularly, in the Russian Federation; thus there is an urgent need to formulate oral policy and program, which the country currently lacks in.

## Background

Oral health is a major component of public health as it affects the overall health and well-being of the person throughout life [1]. But, very few countries have implemented oral health programs [2]. WHO defines oral health as “a state of being free from mouth and facial pain, oral and throat cancer, oral infection and sores, periodontal (gum) disease, tooth decay, tooth loss, and other diseases and disorders that limit an individual’s capacity in biting, chewing, smiling, speaking, and psychosocial wellbeing [3].” The most common oral problems include dental cavities, periodontal diseases, oral cancer, oral infectious diseases, trauma from injuries and hereditary lesions [3]. Notably, oral problems are hidden and invisible and so known as ‘neglected epidemic’ [2, 4]. The literature suggests that several socioeconomic, cultural and environmental conditions increase the risk of oral problems [4]. The studies from different settings have shown that the risk of oral problems was significantly higher among adults with diabetes, hypertension and coronary heart disease (CVD) [5–7]. The other studies also reported that education, age [8] ethnicity, religion, socioeconomic status [9], gender [10], body mass index (BMI) [11], smoking [12], alcohol use [13] are important risk indicators of oral problems.

Oral health problems including dental caries, periodontitis, and oral cancers are a major concern especially in developing countries [14]. According to the Global burden of disease (GBD) 2016 study, worldwide oral problems caused 19 million years lived with disability (YLD) in 2016 [15]. The Institute of Health Metrics and Evaluation (IHME) data indicate that oral problems have been one of top ten causes of YLD in Brazil in the last decade [16]. The estimates suggest that the rate of periodontal health was 12.6% in middle-aged and older adults in China [17]. Similarly, 90% of the adult population had periodontitis in India [18]. Other estimates indicate that 36% adults had any oral problem in South Africa [19]. But to our best knowledge, none of the studies have examined the prevalence of oral problems and associated risk indicators using large-scale data in particularly in BRICS countries. Thus, there is a need to examine the epidemiology of oral problems, especially in adults using large-scale population representative household survey data. To fulfill the purpose, we used the data from the first round of Study of Global AGEing and Adult Health (SAGE) conducted by WHO in 2007–10 in six lower and upper-middle-income countries China, Ghana, India, Mexico, the Russian Federation, and South Africa. The study was not conducted in Brazil [20]. Although SAGE was conducted in South Africa, we could find very few cases of oral problems in South Africa (2%). Hence our analysis includes the Russian Federation, India, and China only from BRICS countries.

The Russian Federation, India, and China make useful comparisons as they contrast in geography, population, races and ethnic groups [21]. While the Russian Federation is the geographically largest country in the world and represents developed economy, India and China are the most populous countries representing developing economies. The Russian Federation and China are ahead of India in terms of demographic and epidemiological transition. In recent years, a simultaneous rapid economic growth is seen in all countries [22]. The health care system in these countries is mixed in which government plays a major role in terms of service delivery [23]. The average life expectancy for both sexes at birth in the Russian Federation, India, and China are 70, 68, 76 years respectively in 2014 [24].

The current expenditure on health as % of GDP was 5.3% in the Russian Federation, 3.7% in India (lowest in BRICS countries) and 5.0% in China in 2016 [25]. While all citizens are covered under mandatory health insurance (MHI) in the Russian Federation, universal health insurance covered about 95% of the population in China in 2011 [26, 27]. The health insurance is voluntary in India with very low coverage (17%) [28]. The out-of-pocket health expenditure was 40, 65, 36 % of current  expenditure on health in the Russian Federation, India, and China respectively in 2016 [29]. Having said that, the present study examines the prevalence of oral problems in adults in selected BRICS countries – the Russian Federation, India, and China. Our study also investigates the risk indicators associated with oral problems.

## Methods

### Study design and setting

We used data from the first round of Study of Global AGEing and Adult Health (SAGE) conducted by WHO in 2007–10. SAGE is a national longitudinal study in six lower and upper-middle-income countries China, Ghana, India, Mexico, the Russian Federation, and South Africa. The study includes nationally representative samples of adults aged 50+ years with comparative samples of adults aged 18–49 years. SAGE is aimed to generate valid, reliable and comparable information on a range of health and well-being outcomes of public health importance, in adult and older adult populations. The sampling frame included a list of villages in rural areas and Census Enumeration Blocks (CEB)/wards in urban areas.

It adopted a multistage cluster sampling design in all countries, two-stage sampling in rural areas and three-stage sampling in urban areas. The SAGE collected data using standardized questionnaires (country specific adaptions) including self-reported and objective health measures (performance tests, anthropometry, and biomarkers). SAGE collected information on self-reported morbidities and health conditions based on interviews, anthropometric and health measurements, and blood tests. SAGE surveys interviewed 30,376 adults (18 years and above) including 4335 adults in the Russian Federation, 11,230 in India, and 14,811 in China [20]. Since we excluded adults whose anthropometric measurements are not available in SAGE, our analytical sample sizes are 3692, 10,914 and 13,827 adults in the Russian Federation, India, and China respectively.

### Ethics Statement

The study is based on a publically available dataset, Study of Global AGEing and Adult Health (SAGE), which does not have any identifiable information on survey participants. One can access the data with prior permission from http://apps.who.int/healthinfo/systems/surveydata/index.php/catalog/sage for research purpose. SAGE strictly followed all ethical concerns including informed consent. Hence, no ethical approval or informed consent is needed for the present study.

## Variables

### Dependent variable

The dependent variable is the oral problems. The SAGE asked adults, “During the last 12 months, have you had any problems with your mouth and/or teeth, including problems with swallowing?” Hence oral problems are defined in SAGE as if an adult had any mouth and/or teeth related problems including swallowing problems in last 1 year of the survey. The outcome variable is binary, so we coded it as ‘1’ if s/he had the oral problems and as ‘0’ otherwise.

### Independent variables

**Fig. 1:**
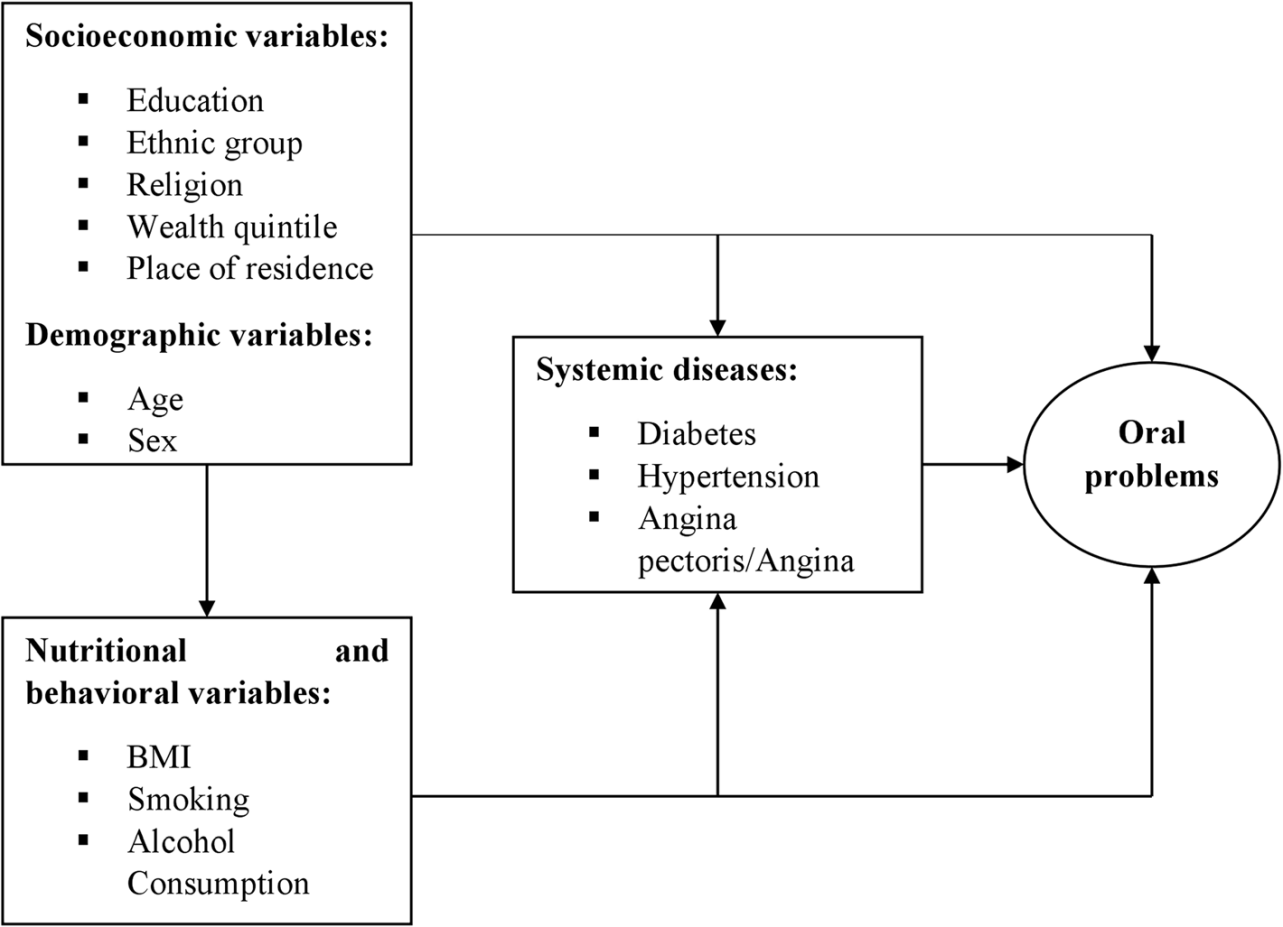
Hierarchical model of indicators associated with oral problems in adults, The Russian Federation, India and China

Based on previous studies, we included several independent variables in the analysis [4–13]. Further, we hierarchically categorized them on the following three groups (1) socioeconomic and demographic variables (2) nutritional and behavioral variables and (3) systemic diseases. We designed the conceptual and theoretical model and used it as the basis for the grouping of variables. The hypothesized model assumes that risk indicators can hierarchically affect oral problems through the variables kept at subsequent levels. (Fig. 1).

### Socioeconomic and demographic variables (Group 1)

Education (completed up to secondary, completed higher secondary and above), country-specific ethnic group and religion, wealth quintile (poorest, poorer, middle, richer, richest), place of residence (urban, rural), age of adults in years (18–44, 45–59, 60 and above), and sex of the adult (male, female).

### Nutritional and behavioral variables (Group 2)

BMI (< 25 kg/m^2^, ≥ 25 kg/m^2^), smoking (yes, no), and alcohol consumption (yes, no).

### Systemic diseases (Group 3)

The systemic diseases include diabetes, hypertension, and angina pectoris/angina. In SAGE, following questions were asked from adults to get information on those diseases:

1. Have you ever been diagnosed with diabetes (high blood sugar)?
2. Have you ever been diagnosed with high blood pressure (hypertension)?
3. Have you ever been diagnosed with angina or angina pectoris (a heart disease)?

All systemic diseases were coded into dummy variables. Those who reported ‘yes’ were coded as ‘1’ and rest were coded as ‘0’.

### Statistical Analysis

We calculated the mean age of adults who had the oral problems in all three countries and used a t-test for its comparison. We also estimated the prevalence of oral problems in adults shown as the proportion of adults who had oral problems in the past 12 months preceding the survey. Finally, we used a multivariable binary logistic regression model to identify the risk indicators of the oral problems in selected BRICS countries. In the regression model, first we included socioeconomic and demographic variables because they can influence all variables on subsequent levels in the hierarchical conceptual model. Then we included nutritional and behavioral variables, which may mediate the association between socioeconomic and demographic variables and oral problems. The systemic diseases were included at the last in regression model as the probable risk indicators of oral problems that can be influenced by all variables in the previous levels. The analysis was carried out in STATA 13.0. Country-specific appropriate sampling weights were used in the estimations.

## Results

**Fig. 2:**
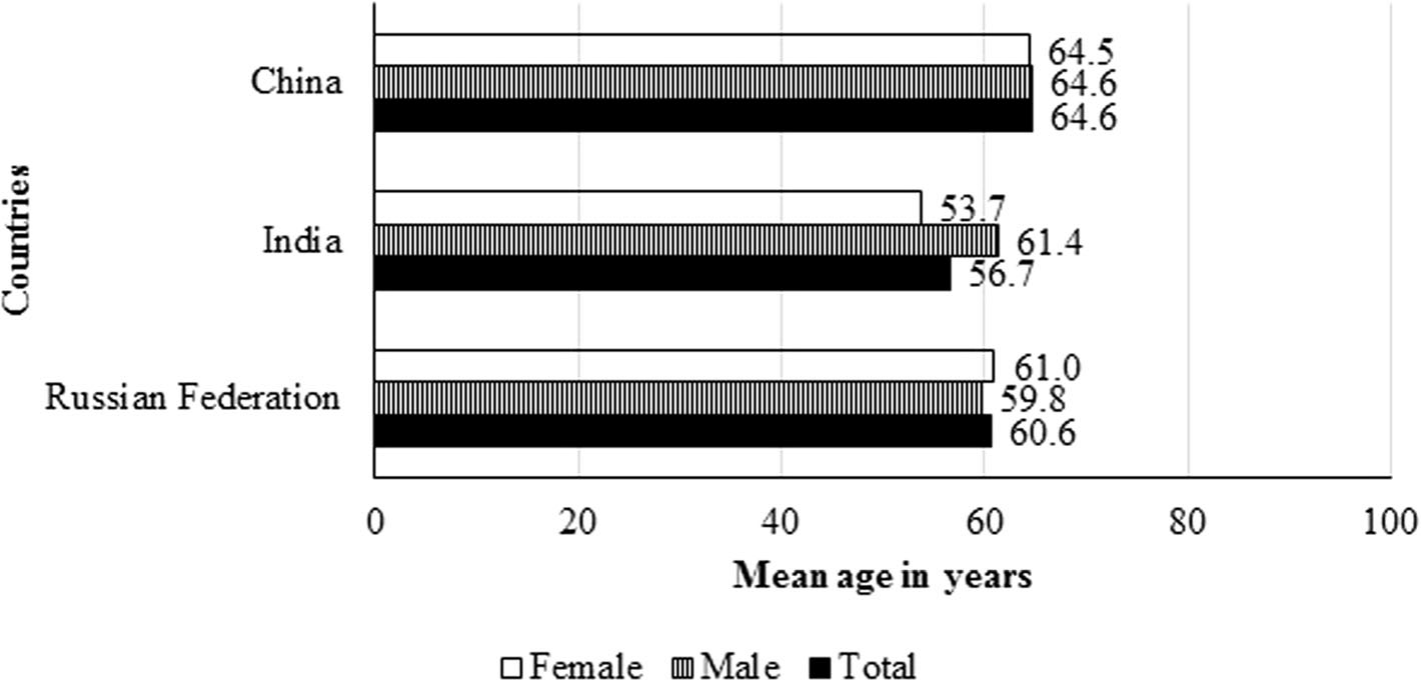
Mean age of adults who had oral problems, The Russian Federation, India and China

The mean age of adults who had oral problems was higher than 50 years in all the three countries considered in the analysis. The mean age was lowest in India (57 years; SD: 15) and highest in China (65 years; SD: 11). The mean age did not vary by sex of adults in the Russian Federation and China. But, it was significantly higher for males (61 years; SD: 13) than females (54 years; SD: 16) in India (Fig. 2).

**Fig. 3:**
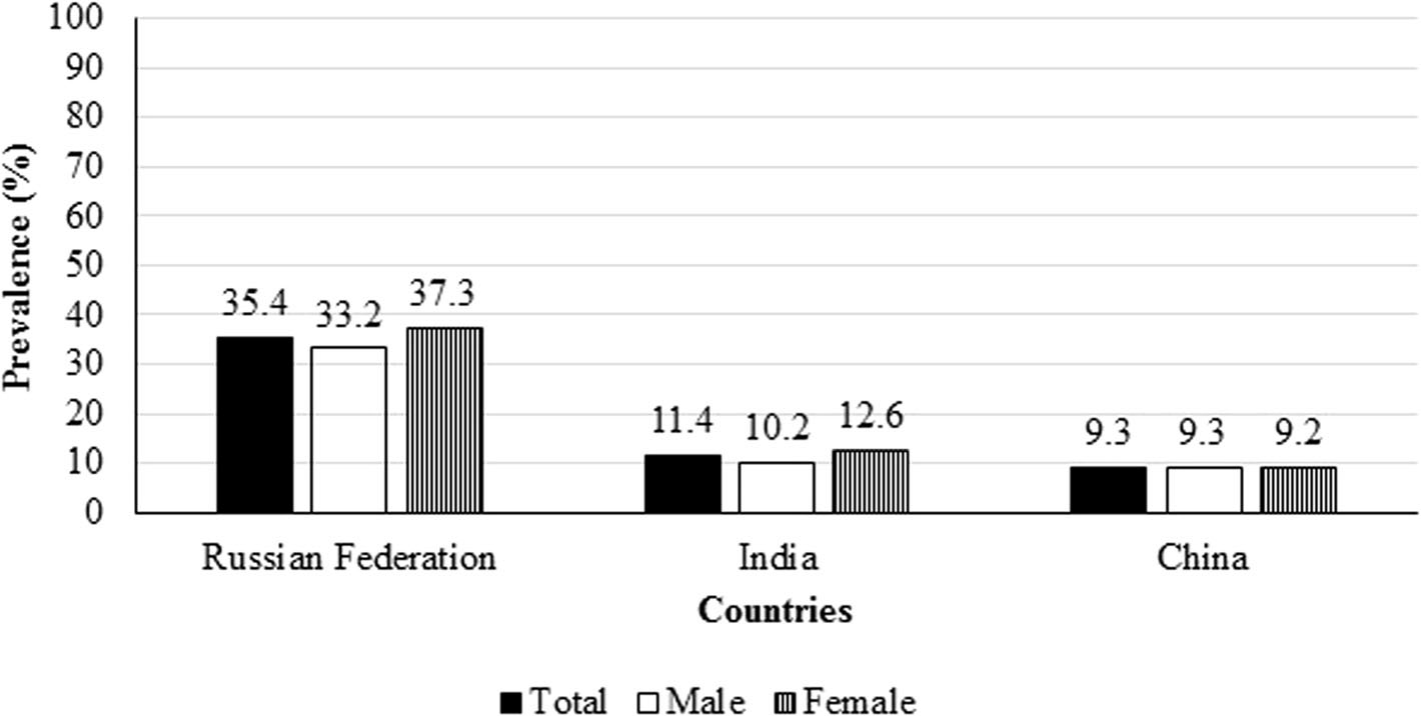
Prevalence of oral problems in adults by sex, The Russian Federation, India and China. Note: Prevalence of oral problems is the proportion of adults who had oral problems in past 12 months preceding the SAGE survey

The prevalence of oral problems varied considerably across the three countries. The highest and lowest prevalence was in the Russian Federation (35%; 95% CI: 30.0–46.2) and China (9%; 95% CI: 6.7–12.6) respectively. The oral problems were slightly higher in females than males in the Russian Federation and India (Fig. 3).

The prevalence of oral problems increased with increase in the education level of adults in the Russian Federation. The oral problems in adults varied by ethnic group, religion, and wealth quintile in all three countries. The prevalence of oral problems was higher in adults residing in urban areas in the Russian Federation and rural areas in India and China. The highest prevalence of oral problems was in adults aged 60 years and above in India and China. The oral problems were higher in adults having BMI < 25 kg/m^2^, smokers, adults using alcohol in the Russian Federation and only in adults using alcohol in China. The prevalence of oral problems was higher in adults with diabetes, hypertension, and angina pectoris/angina in India and China. (Table 1).

**Table 1:** Prevalence of oral problems in adults by socioeconomic and demographic, nutritional and behavioral variables and systemic diseases, The Russian Federation, India and China

Socioeconomic and demographic, nutritional and behavioral variables and systemic diseases	Russian Federation		India		China	
	%	*N* = 3692	%	*N* = 10,914	%	*N* = 13,827
Socioeconomic and demographic variables						
Education						
Completed higher secondary and above	38.9	3215	7.9	2539	6.1	3967
Completed up to secondary	12.0	477	12.5	8375	10.6	9783
Ethnic group						
Russian	36.2	2910	NA	NA	NA	NA
SC and ST	NA	NA	10.0	2809	NA	NA
Han	NA	NA	NA	NA	9.4	13,640
Other	36.1	574	11.9	8062	1.8	156
Religion^a^						
Main	37.4	2692	11.1	1725	9.1	818
Other	30.3	1000	12.9	9189	11.2	12,921
Wealth quintile						
Poorest	28.8	460	13.0	2399	11.0	1381
Poorer	30.8	464	10.1	2328	7.6	2268
Middle	21.3	509	12.8	2203	6.2	2575
Richer	32.5	927	11.5	1926	13.3	3248
Richest	46.8	1328	9.3	2058	8.4	4308
Place of residence						
Rural	19.8	707	11.8	8154	13.1	7268
Urban	39.2	2985	10.2	2760	5.0	6559
Age of adult in years						
18–44	48.7	1776	8.2	6814	5.0	6800
45–59	28.4	1104	13.5	2737	12.4	4961
≥ 60	16.1	812	23.1	1364	16.0	1970
Sex of adult						
Male	33.2	1671	10.2	5594	9.3	7140
Female	37.3	2021	12.6	5320	9.2	6619
Nutritional and behavioral variables						
Body mass index						
≥ 25.5	33.8	2108	10.6	1274	9.2	4420
< 25.5	37.7	1584	11.5	9640	9.3	9407
Currently smoking						
No	29.5	976	11.3	6250	9.2	9297
Yes	37.6	2716	11.6	4664	9.3	4530
Currently using alcohol						
No	23.8	1708	11.4	9946	8.5	10,159
Yes	45.4	1984	11.0	968	11.4	3668
Systemic diseases						
Diabetes						
No	36.0	3562	11.2	10,569	9.2	13,400
Yes	19.1	130	18.4	345	11.9	392
Hypertension						
No	38.3	2701	11.0	9886	8.9	12,148
Yes	27.7	991	15.4	1028	12.7	1553
Angina pectoris/Angina						
No	36.9	3095	11.2	10,576	9.0	13,274
Yes	27.9	597	18.7	338	17.4	516
Total	35.4	3692	11.4	10,914	9.3	13,827

Total may not match in some cases due to missing information

All values in the table represent absolute numbers and percentages unless otherwise stated. Percentages and Numbers are weighted

*Abbreviation*: *SC* Scheduled Caste, *ST* Scheduled Tribe, *NA* Not Applicable

^a^termed as main if followed in the majority of households in particular country (Christian in Russian Federation, Hindu in India, and None in China)

The results of multivariable binary logistic regression showing the risk of oral problems in adults are shown in Table 2. Age was statistically associated with oral problems in India and China. The adults age 45–59 years were 1.7–2.3 times (95% CI: 1.5–3.2, *p*-value < 0.05) as likely as adults age 18–44 years to have the oral problems. Likewise, the adults age 60 years or more were 2.9–4.2 times (95% CI: 2.5–5.8, p-value < 0.05) as likely as adults age 18–44 years to have the oral problems. Sex was statistically associated with the oral problems in all three countries. Females were 1.3–1.6 times (95% CI: 1.1–2.0, *p*-value < 0.05) as likely as males to have the oral problems.

**Table 2:** Results of multivariable binary logistic regression showing the risk of oral problems in adults, The Russian Federation, India and China

Demographic, nutritional and behavioral variables and systemic diseases	The Russian Federation	India	China
	*N* = 3666	N = 10,871	N = 13,326
	AOR (95% CI)	AOR (95% CI)	AOR (95% CI)
Demographic variables			
Age of adult in years			
18–44	Ref	Ref	Ref
45–59	0.89 (0.66–1.19)	1.72^a^ (1.48–2.00)	2.34^a^ (1.71–3.20)
≥ 60	0.75 (0.55–1.03)	2.94^a^ (2.54–3.40)	4.25^a^ (3.11–5.81)
Sex of adult			
Male	Ref	Ref	Ref
Female	1.64^a^ (1.36–1.98)	1.27^a^ (1.10–1.46)	1.23^a^ (1.08–1.40)
Nutritional and behavioral variables			
Body mass index			
≥ 25	Ref	Ref	Ref
< 25	0.98 (0.82–1.18)	1.12 (0.95–1.35)	1.31^a^ (1.16–1.48)
Currently smoking			
No	Ref	Ref	Ref
Yes	1.09 (0.88–1.37)	1.07 (0.95–1.21)	1.11 (0.96–1.29)
Currently using alcohol			
No	Ref	Ref	Ref
Yes	1.85^a^ (1.56–2.19)	0.85 (0.67–1.08)	1.18^a^ (1.03–1.36)
Systemic diseases			
Diabetes			
No	Ref	Ref	Ref
Yes	1.18 (0.90–1.54)	1.29^a^ (1.04–1.63)	1.14 (0.92–1.42)
Hypertension			
No	Ref	Ref	Ref
Yes	1.31^a^ (1.11–1.56)	1.28^a^ (1.10–1.49)	1.12 (0.98–1.27)
Angina pectoris/Angina			
No	Ref	Ref	Ref
Yes	1.24^a^ (1.04–1.49)	1.47^a^ (1.15–1.87)	1.01 (0.83–1.23)

Regression model is adjusted for all socioeconomic and demographic, nutritional and behavioral variables and systemic diseases, but results are not shown for socioeconomic variables due to insignificant findings

*Abbreviation*: *AOR* adjusted odds ratio, *CI* confidence interval, *Ref* reference category

^a^significant at 5% level

BMI was statistically associated with oral problems in adults only in China. The adults having BMI < 25 kg/m^2^ were 1.3 times (95% CI: 1.1–1.5, *p*-value < 0.05) as likely as those having BMI ≥ 25 kg/m^2^ to have the oral problems. Alcohol consumption was statistically associated with oral problems in the Russian Federation and China. Adults using alcohol were 1.2–1.8 (95% CI: 1.0–2.2, *p*-value < 0.05) times as likely as those not using alcohol to have the oral problems. Smoking was not associated with oral problems in any of the countries considered in the analysis.

Hypertension and angina pectoris/angina were statistically associated with oral problems in the Russian Federation. Adults having hypertension were 1.3 times (95% CI: 1.1–1.6, *p*-value < 0.05) as likely to have the oral problems as their counterparts. Likewise, adults having angina pectoris/angina were 1.2 times (95% CI: 1.0–1.5, p-value < 0.05) as likely to have the oral problems that adults who did not have angina pectoris/angina. In India, adults having diabetes, hypertension, and angina pectoris/angina are 1.3–1.5 times (95% CI: 1.0–1.9, p-value < 0.05) as likely as adults not having these diseases to have the oral problems.

## Discussion

Our study perhaps is the first, which examines the prevalence of oral problems in adults in selected BRICS countries. Also, it is the first study that investigates the risk indicators of the oral problems in adults using large-scale nationally representative population-based data. The mean age of adults who had oral problems varies between 57 years in India and 65 years in the Russian Federation. Also, the significant male-female gap is seen in the mean age in India. However, the mean age of adults who had oral problems is almost similar in both sexes in the Russian Federation and China.

We found the prevalence of oral problems was lowest in China and the highest in the Russian Federation. Also, socioeconomic and demographic, nutritional and behavioral indicators and systemic diseases affect the prevalence of oral problems in adults in BRICS countries. In multivariable binary logistic regression BMI, age, sex, alcohol consumption, diabetes, hypertension, and angina pectoris/angina are significantly associated with oral problems. Adults with BMI less than 25 kg/m^2^, diabetes, hypertension and, angina pectoris/angina are more likely to have oral problems. Female adults and adults using alcohol are also more likely to have oral problems than their counterparts in BRICS countries. Our findings are consistent with findings of previous studies from various parts of the world [5–8, 11, 12, 30].

It is important to discuss the existing oral health care delivery system in the selected BRICS countries. The oral health care system is mixed in the Russian Federation, India, and China and private providers are major players in delivering oral health care services [14, 26, 31]. It is interesting to note that the Russian Federation has neither an oral health policy nor a national strategy or program for oral health. Oral health care facilities are mostly in urban areas, and rural population has to travel a considerable distance to access oral care. Moreover, oral facility network and volume of health care in municipal and state hospitals have declined in the Russian Federation since the year 2000. The coverage of preventive oral health examination has also decreased by 30% for adults [26].

In India, oral health policy is an integral part of the National Health Policy since the year 1995 [32]. However, there is a growing demand for separate oral health policy in the country [18]. India also has a National Oral Health Program (NOHP) in action under the National Health Mission (NHM) [33]. Even though oral health is a component of primary health care in the country; oral care services are available at primary health care centers (PHC) in a very few states [14]. Also, dentist or dental public health (DPH) professionals are available at less than 20% of PHCs in rural areas [34]. Besides, oral health facilities in India are mostly concentrated in urban areas. The ideal WHO dentist population ratio is 1:7500 for all countries [35]. But, this ratio is highly uneven in India, and it is 1:10000 in urban areas and 1:150000 in rural areas [14]. The recently conducted National Family Health Survey 2015–16 (NFHS-4) in India reports a significant urban-rural gap in women age 15–49 have ever had an oral cavity examination. Overall only 12% of women age 15–49 in India had ever undergone oral cavity examination [36].

China has its oral health policy. The National Health and Family Planning Commission (NHFPC) has been instrumental in developing oral disease control and prevention strategy. The Ministry of Health conducted four rounds of the National Oral Health Survey (NOHS) in 1983, 1995, 2005 and 2015 [31, 37]. But, China is also not far behind the Russian Federation and India, where the distribution of oral health facilities is unbalanced. There were 253 stomatological tertiary care hospitals in urban areas compared to only 64 in rural areas in 2011. Interestingly, the dentist population ratio was 1:15000, almost double than that of ideal WHO ratio [31].

Our study findings have important policy implications. Given the striking prevalence of oral problems in the Russian Federation, the country must immediately formulate oral health policy and program. The Russian Federation might learn from the experiences of China for controlling the high burden of oral problems in the country. The oral problems are significantly higher in females in the selected countries and adults consuming alcohol in the Russian Federation and China. Notably, alcohol use in the Russian Federation has always remained the highest in the world [38]. Also, the alcohol-related health problems are on the rise in China since 1980 [39]. Hence, the oral health program must target females and people consuming alcohol in the Russian Federation and China.

Our findings lend support to the demand for separate oral health policy in India. Like China, oral health screening programs must be started on a regular basis for early detection and timely diagnosis of an oral problem. India, in particular, must strengthen the existing oral health care delivery system, and steps must be taken to balance the dentist-population ratio particularly in rural areas. There is also a need for Information Education Communication (IEC) and Behavior Change Communication (BCC) in India in general and rural areas in particular.

### Limitations of the study

There are a few limitations in the study. The SAGE data used in the study dates back to 2007–10. However, SAGE is the most recent dataset that has collected information on the oral problem in four of the 5 BRICS countries. The second limitation is the self-reporting of oral problems in the study. But self-reported methods are widely used in large-scale population-based surveys, and many studies confirmed the reliability and validity of self-reporting of health-related events [40, 41]. In particularly, a literature review of 19 studies also concluded that there was acceptable validity for self-reporting of oral health outcomes [42]. Third, we could not differentiate oral problems into dental caries, periodontitis, and oral cancer because SAGE does not provide such detailed information. Strikingly, SAGE collects separate information on edentulism and does not include in oral problems. Fourth, we found a significant statistical association between hypertension and angina pectoris/angina and oral problems. However, some studies have shown that oral problems increase the risk of hypertension and angina pectoris/angina [43–45]. We have not examined this dimension in the analysis. Fifth, we could not adjust our results for the effect of sugar, cool-drink as SAGE does not collect such information. Sixth, the recall time for oral problems is 12 months. Such a recall period may seem large to some. However, all SAGE surveys conducted by the WHO uses a 12-month recall period to measure the prevalence of oral problems. It can also be argued that adults who have challenges with short or long term memories may not be able to recall oral problems in the last 12 months. But it will be interesting to note there was a short set of questions for adults aged 50 and above about memory preceded the main set of questions in the individual questionnaire to determine whether the respondent was cognitively or physically competent to interview in SAGE. In case, interviewer were allowed to conduct a proxy interview (who knew the respondent well and was able to accurately answer questions about the respondent’s health and well-being on behalf) [46–48]. However, the proxy interviews were conducted for only 2% respondents in China [48]. Strikingly, the results of sensitivity analysis showed that the prevalence of oral problems did not vary with and without the inclusion of proxy respondents in China. Seventh, we could not carry out the analysis for South Africa as only 2% of the respondents reported oral problems in South Africa. However, the existing studies suggest better oral health situation in South Africa in terms of the WHO classification. At least three oral health surveys have been carried out in South Africa in the last three decades [49].

## Conclusions

Despite these limitations, the study brings to the forefront the high burden of oral problems in adults in selected BRICS countries where oral diseases have become more prevalent in the last decade. The study also identifies the important risk indicators of oral problems in adults. Our study raises a number of questions on the existing oral health care delivery system in selected BRICS countries. The oral health program and strategies must be redesigned to bring down the burden of oral diseases.

## Data Availability

The SAGE data is publically available and can be assessed for research purpose with prior permission from http://apps.who.int/healthinfo/systems/surveydata/index.php/catalog/sage.

## Abbreviations

BCC: Behavior change communication
BMI: Body mass index
BRICS: Brazil, The Russian Federation, India, China, South Africa
CEB: Census enumeration block
CVD: Cardiovascular diseases
DPH: Dental public health
GBD: Global burden of disease
GDP: Gross domestic product
IEC: Information education communication
IHME: Institute for Health Metrics and Evolution
MHI: Mandatory health insurance
NFHS: National family health survey
NHFPC: National health and family planning commission
NOHP: National oral health program
PHC: Primary health center
SAGE: Study of global ageing and adult health
WHO: World health organization
YLD: Years lived with disability
